# Artificial Intelligence inspired methods for the allocation of common goods and services

**DOI:** 10.1371/journal.pone.0257399

**Published:** 2021-09-29

**Authors:** Spyridon Samothrakis

**Affiliations:** Institute for Analytics and Data Science, University of Essex, Colchester, Essex, United Kingdom; University of Almeria, SPAIN

## Abstract

The debate over the optimal way of allocating societal surplus (i.e. products and services) has been raging, in one form or another, practically forever; following the collapse of the Soviet Union in 1991, the market has taken the lead vs the public sector to do this. Working within the tradition of Marx, Leontief, Beer and Cockshott, we propose what we deem an automated planning system that aims to operate on unit level (e.g., factories and citizens), rather than on aggregate demand and sectors. We explain why it is both a viable and desirable alternative to current market conditions and position our solution within current societal structures. Our experiments show that it would be trivial to plan for up to 50K industrial goods and 5K final goods in commodity hardware. Our approach bridges the gap between traditional planning methods and modern AI planning, opening up venues for further research.

## 1 Introduction

The historical experience of the late 20th century brought the market to the forefront of societal organisation. A sequence of events, which includes the collapse of the Soviet Union, the liberal turns in the UK and US and China’s turn to the market under Deng Xioping, made it clear that all policy (if any) was to be enacted through markets. The “calculation debate” [1, 2], an open discussion about central (economic) planning vs markets, was resolved; if humanity was to prosper, the state would have to exercise (at best) a very limited control over market mechanisms. The demise of economic planning took with it the utopia imperative; grand, state sponsored, schemes to improve the human condition were judged as inherently flawed [3], resulting in more pain than anything, so they were better avoided. This backlash was not completely unjustified; planning, as a technical term, refers to a process where a machine/group of people spends time “thinking” really hard about the feature and identifies a sequence of actions that would lead to long term “happyness”. Once this sequence of actions is discovered, it is executed in the real world. In terms of economics, the actions can be conceptualised as what, when, how etc. to produce goods and services. Without the use of computers and smart algorithms, the shortcuts one would need to take are simply too crude, and along with certain political imperatives, resulted in serious economic problems [4]. At the very same time that in economic sciences planning was ostracised, in Artificial Intelligence (AI) planning (using a similar framework as economic planning, but different substrates) saw a tremendous renaissance, following the wider upheaval of the whole field. We can now create super-human game (e.g. chess, go) players in artificial environments using a variety of planning methods [5].

With the collapse of state volition for economic planning, it is no surprise that research in alternatives (or partial alternatives) to the market remained very limited in scope. In this paper, we revisit one such alternative paradigm of societal distribution, whose invention (or inspiration) goes back quite some time [6–8]. We will provide a base for removing certain products from market circulation and provision them directly to citizens. The calculation of using products and services directly is generally called “planning in natura”[9], and has direct links to Universal Basic Services. The goal of planning methods is to remove the anarchy (and uncertainty) of production and provide citizens with consumption guarantees. Contrary to most of the authors we cite, our ambitions are somewhat social-democratic. We do not aim to replace the market, but instead focus on removing human reproduction from strictly ideological mechanisms. In fact, a conservative government not “tied” to market ideology could easily start implementing such a programme. The goal of our specific programme is to match citizens and production units directly while monitoring the plan as closely as possible—in order to take corrective action—on a daily basis. Plan goals are to be formed using data collected from production units and citizens. We are not aware of any methods that attempt to plan production on the individual level, nor has there ever been an automated way to monitor the plan or amend it using data—though other efforts point to similar direction [10, 11]. Conceptually, *our major contribution is a direct link between AI planning (i.e. MDPs) and traditional input-output tables, thus allowing to bring forth the power of modern AI methods to traditional economic planning problems*. The closest a quasi-automated system of planning that reached an (partial) operational level was Project Cybersyn [12], but this was dismantled in a hurry following Pinochet’s coup. Within the Soviet Union there is evidence that planning from final demand was seen as a “bourgeois” [13] and was never allowed, leaving production planning to the level of industrial goods (e.g., steel). The insistence to create plans and the focus of soviet economy to “build machines that build machines” might have contributed to the grim life of the soviet citizens in terms of consumer products. Prior to the late 1970s, when the demise of USSR became evident, some form of planning was always accepted within capitalist societies [14]. Japanese economists were effectively trained in planning by explicitly going through the works of Marx [15] until the late 80s.

The rest of the paper is organised as follows; in Section 2 we provide a generic discussion on the background and debate between economic planning and market economics, but also nudge at the link between economic planning, reinforcement learning and AI planning. Section 3 introduces a new model, which we term Open Loop In Natura Economic Planning. In Section 4 we discuss data collection issues—and generally re-think the problem from the point of view of individual production units and citizens, while in Section 5 we perform a series of simulations. We discuss limitations in Section 6; we conclude with a short discussion in Section 7.

## 2 Background

### 2.1 The state and social democracy

Following the second world war, a large effort to direct the output of national economies was set in motion. Social democratic and labour parties, reinvigorated in popularity by the horrors of war, set ambitious programmes of state provision, commonly referred to as “democratic economic planning”. To quote Sir Stafford Cripps, in his position as the UK’s Chancellor of Exchequer, who, when discussing democratic planning claimed that [16] “*…we are out after something a great deal more important than a good piece of planning machinery or even than a particular way of organising our industries and services. Our aim is to create a Happy Country in which there is equality of opportunity…*”. A set of industries was nationalised (including the banks, coal, telecommunications, gas, electricity, public health etc)—for the case of the UK see [17]. This was roughly the consensus, respected by conservative governments worldwide, that most of the world has followed until almost the 1980s. From that point onward we see a reversal of the state intervention trend and widespread privatisation. Though there is still a debate as to why this happened, from the electoral perspective one can observe the collapse of social democratic parties owing to the breaking down of the electoral coalitions between liberal elements and the working class (see [18] for a thorough discussion). The reversal of the trend brought widespread privatisation and the re-introduction of the market. This process of “re-marketisation” went by different speeds in different countries and different economic sectors, but arguably the process is still ongoing. Even when certain services are still nominally free at the point of use (mostly in healthcare), the vast majority of utilities (including education) is slowly moving to fee-paying models and internal markets. The victory of the market is so absolute that certain authors complain in the popular imagination: “it is easier to envision the end of the world than the end of capitalism” [19].

### 2.2 Socialist planning in actually existing socialism

While capitalist counties were moving away from the social-democratic model, the end of historically existing socialism lead to the introduction of “shock therapies”[20] and widespread, fast, privatisation, with at the very least questionable results. When privatisation did take a more structured form, as in the case of China, state planning was replaced due to associations with poverty. Quoting [21]*“…one of our shortcomings after the founding of the People’s Republic was that we didn’t pay enough attention to developing the productive forces. Socialism means eliminating poverty. Pauperism is not socialism, still less communism.”* The state took a back sit into acting as planner and started using financial means to measure (and drive) success. Fiver-year plans no longer meant exact outputs, but rather strategic visions [22], with specific GDP per capita’s aims, akin to industrial strategies elsewhere. This failure of planning, can, at least partially, be attributed to practical factors. Computers and algorithms of the scale required to plan effectively did not exist a the time, and when the first thoughts of such projects where entertained (e.g. see [23]) they were not supported adequately. Indeed, if there is anything to be said is that it is almost a miracle that any form of state planning was attempted given the means available.

### 2.3 Why planning?

Von Mises and Hayek [1], writing in the height of socialist revolutions, started putting together a critique of socialism, and more specifically (economic) planning. Parts of their critique (and this of their successors) sound still valid—for example same of their points on Marx’s treatment of skilled vs unskilled labour. Here we will concentrate on the arguments of planning using products and services (i.e. 10 kilos of rice, 20 pounds of flesh, 10 hours of electric supply) vs a market price allocation mechanism. Whether an optimal (automated or not) planner of such type could even exist is termed the *calculation debate*. Arguments against the existence of an optimal planning mechanism fall into different camps, with some being aligned to moral questions (“it is unfair to just allocate goods” or “it is undemocratic”), computational (“you can’t compute the intermediate goods to produce”) or epistemic (“there is no way for the planner to know what to produce”). We will not discuss the democratic issue in this paper, though we strongly feel that the market is exceptionally undemocratic. It is now accepted by even the opponents of planning that computation should not be an issue [24]. The epistemic argument, which is still very valid, entails that an optimal planner would not know *what* to compute. A price mechanism would allow whoever is engaged with the market to express their preferences of goods in terms of how much they would be willing to pay, i.e. a very subjective preference function. Prices that (for producers) might, for example, depend on the availability of goods [25]. In its extreme this holds true for consumers, as we have seen examples of iPad-for-kidney selling [26], though we think it is safe to class such behaviours as pathological. If one makes the assumption of truly subjective values that vary continuously and are also widely different from person to person, then indeed a market might be able to allocate surpluses somewhat better than a plan. However, if you do accept that the majority of the population shares some similar preference function, at least in their top priorities (e.g. food, shelter, basic communication devices, electricity, health), the argument is nonsensical and applies only to incorporeal beings. Insofar as there are relatively slow changing patterns in consumption, standard machine learning models, combined with one’s own predictions can be used to forecast demand.

### 2.4 Why not alternative forms of market organisation?

A popular counterargument against planning is one of efficiency, quite often expressed in macro-economic aggregates (e.g. GDP growth, the gini coefficient). These tend to hide vast complexities of the underlying tendencies of the system. In the game-theoretic literature (which is closely aligned to economic models), different notions of where a system should equilibrate in terms of specific agent rewards have been explored and unpacked. In effect, these try to predict where a large population of agents would end up, if left to explore and learn freely, given imposed game rules. For example, [27] provide four types of correlated equlibria: utilitarian (which maximise the sum of rewards for all agents), plutocratic (which maximise the maximum reward for all agents), dictatorial (which maximise the maximum reward of a specific agent) and egalitarian (which maximise the minimum reward for all agents). Instead of planning, the state could play the role of “traffic lights”, and try to stabilise the whole system by favouring certain equilbria. Arguably, and almost by definition, once a system stabilises to one set of equilibria it is hard for it to move another, as unilateral movement by any agent would is strongly disincentivised. Historically, examples like anti-monopoly laws, taxation, demurrage and the welfare state point to a countermovement towards plutocratic and dictatorial equilibria, as it looks like markets tend to generate pareto distributions [28], i.e. very few individuals tend to accumulate overwhelmingly. Monopolies, as explained by their proponents [29] allow for both innovation and “concentrated application of force”, something that would not be feasible if one a business is surviving day-to-day due to heavy taxation and competition. A modern version of planning should not be seen as a fully centralised top-down-controlled structure, but rather as a game that has egalitarianism baked in and not as an afterthought; alternatively one can see the whole edifice as a decentralised democratic monopoly.

### 2.5 Input-output economics and planning

The problem of planning has been formally defined in [30]. Per unit of time *t*, a set of demands *d* for certain goods (e.g, products, services) are to be satisfied for *c* citizens. The planner’s goal is to satisfy the demand of each citizen. In AI terms, we have something akin to a Markov Decision Process (MDP), with an agent (the planner) receiving information (the state) on the plan and a set of rewards related as to how closely the demand is met.

The parent of modern mechanisms for planning (in this context) is what is termed the input-output model, which is thoroughly reviewed by [31]. The model comprises of an *nxn* Matrix *A* of technical coefficients, a vector *x* of production level (i.e. how much we should produce for each product) and a demand vector *d*. The columns of the coefficient matrix conceptually ask the question “how many units of each good to produce a single good of the type portrayed in this column do we need?”. The dot product of each row with the technical coefficients represents the consumption of a specific good. The demand vector *d* represents how much external demand there is, i.e. that Eq 1 holds:

$$\begin{array}{c} x_{i}=a_{i1}x_{1}+a_{i2}x_{2}+\ldots +a_{in}x_{n}+d_{i} \end{array}$$ (1)

In matrix notation, we have Eq 2:

$$\begin{array}{c} x=Ax+d\Rightarrow (I-A)x=d \end{array}$$ (2)

Something to note here is that traditional input-output models have no notion of time—all production is taking place within the same temporal unit. This is somewhat counterintuitive (and problematic for actual planning), but it allows a first easy approximation. It is the model proposed by [32], covered by [33] and, with further additions (based on linear optimisation) discussed in [9]. Very similar concepts, without a specific target but with the goal of directly maximising output are also discussion by in [23]. With no time element, the model remains suitable for very high level strategic planning—and indeed such models are widely used currently (e.g. most states publish input-output tables using monetary prices). There is also very widespread literature on input-output models, but not with state planning in mind.

## 3 Open Loop In Natura Economic Planning

Our method (Open Loop In Natura Economic Planning—OLIN-EP) builds upon the basic input-output framework. It creates a fundamentally different planning landscape than IO tables and is heavily inspired by current game playing / RL agents. The planning “tick” is no longer a year, but a day, and we expect the plan to be re-calculated based on observations and predictions every night. We no longer operate on abstract notions of aggregate demand, but instead we expect every individual to communicate their demands and projected demands daily. We also expect the productive units to recalculate their input-output coefficients (which we will call IO-coeffs—the values of the matrix *A*) and provide them for plan updates on a daily basis in the form of a function—more on this later. Closing, we maintain a notion of state that is missing from all original formulations. More formally, we operate on an MDP [34] that has the following characteristics:

- Actions x∈A capture what the production *methods, investment profiles and generally actions* that a planner can take to help maximise reward.
- States *s* ∈ *S* capture sufficient statistics of what we want to operate on, as transmitted every morning by production units and citizens. In our case, *s* is simply a goods inventory.
- The transition function *T*(*s*′|*s*, *a*) is formally unknown to us, but it is captured partially by the input-output matrix, partially by the semantics we give to the behaviour of different outputs of the matrix, and it operates on the inventory and externalities.
- The reward function denotes how happy the planner is in a state and is generally encoded as *R*(*s*, *a*). We define later a specific reward function that captures how well the plan targets are met and what damage the plan causes to the world.
- There is a discount factor *γ*, which attenuates closer vs further rewards.

Classic input-output tables are more akin to a Markov Reward Process (MRP), an MDP with no actions. There are no decisions to make; one finds out where the process converges (i.e. how much to produce for each type of good) and tells the industry to produce it. In contrast, we aspire to optimise for production methods, investment etc. The link between MRPs and input-output tables might be not immediately apparent; it stems from a certain method of monte carlo matrix inversions [35] for solving systems of linear equations. Eq 2 can be directly mapped to an MRP, while the addition of production decision (how much to invest, when to invest, what production methods to use) links to MDPs. The extra semantics above add to this framework. We will follow a much simpler (and arguably more inefficient route) in this paper, without converting to an MDP explicitly; for a discussion of the limitations this causes see Section 6.

One can obviously claim that economic planning is more akin to a partially observable MDP (i.e. a POMDP), and this might be true, but unless one is to have the functions that describe the uncertainty over states, there is no reason to do the modelling this way. We could also start acting on histories of states and include externalities and rewards [36], but this might prove computationally infeasible. Claims could also be made that there is strong multi-agent element for the planner—here we assume that everyone involved in the plan has it in their best interest to cooperate.

### 3.1 The model

We adapt a number of innovations to the standard input-output models, by changing the way we position the plan within the economy. As discussed before, the goal of an input-output matrix is to plan for demand at the end of a time period. Since our goal is to provide necessities to sustain humans, we set all “external” demand to zero, and introduce a set of profiles combined with the number of citizens attached to each profile. You can see an example in Table 1. Our input-output matrix describes the interactions between consumption profiles, a set of industrial goods, and a set of final goods. Profiles are columns that describe the allocation of final goods to each citizen that has been assigned this specific profile.

**Table 1:** Our example input-output matrix, for a society of 1300 citizens. Two of the IO-coeffs vary with production levels—as there are three production units (see Fig 1)—the rest are constant. Labour columns are omitted, as all values are zero. There is one industrial good Butter churn and two final goods (Milk and Butter). Demand now just signifies the number of individuals in each profile. Lb is short form for Labour and Prof for profile.

Type	Milk	Butter churn	Butter	Prof 0	Prof 1	Prof Population
Milk	0.001	*f*_01_(*x*_0_)	2.000	3.0	2.0	0
Butter churn	0.000	0.000	*f*_12_(*x*_1_)	0.0	0.0	0
Butter	0.000	0.000	0.000	0.1	0.2	0
Lb(Milk)	0.001	0.000	0.000	0.0	0.0	0
Lb(Butter churn)	0.000	0.012	0.000	0.0	0.0	0
Lb(Butter)	0.000	0.000	0.001	0.0	0.0	0
Profile 0	0.000	0.000	0.000	0.0	0.0	800
Profile 1	0.000	0.000	0.000	0.0	0.0	500

**Fig 1:**
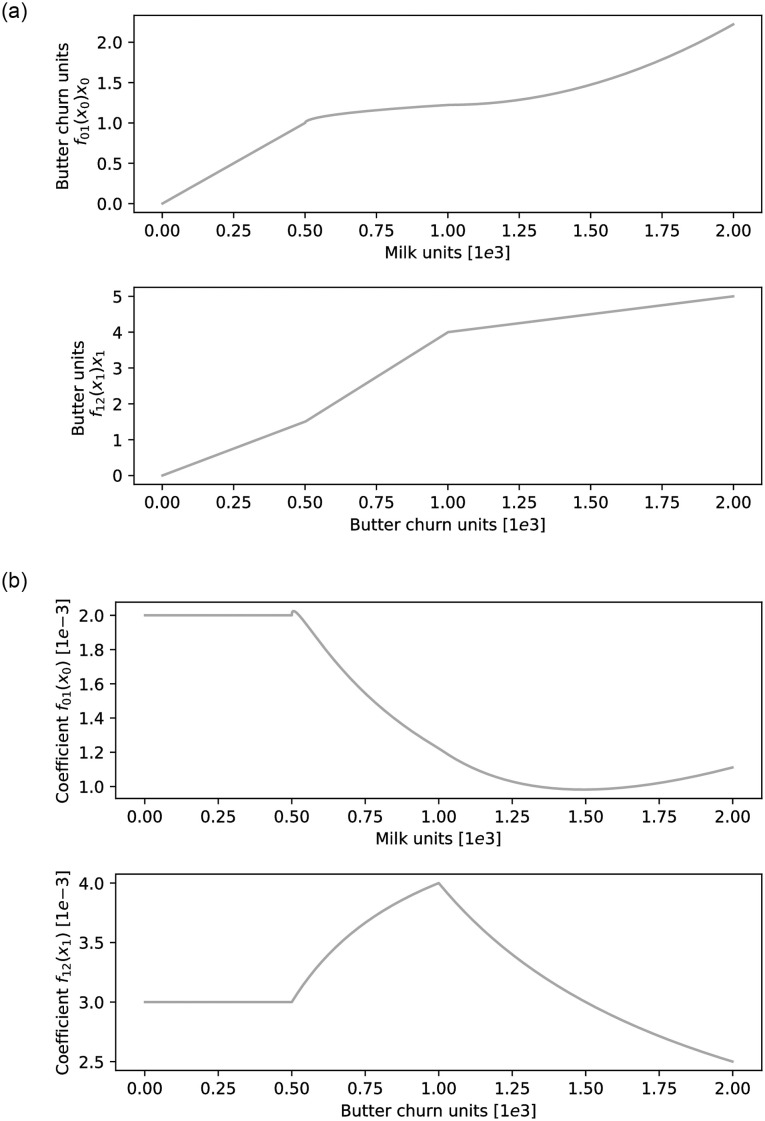
*f*_01_(*x*_0_) and *f*_12_(*x*_1_) derivation from production outputs. There are three fictional production units that follow very different curves in their models. (a) An example of how many units of Milk and Butter churn are needed to create units Butter and Butter churn units as portrayed in the y axis, i.e. *f*_*ij*_(*x*_*i*_)*x*_*i*_. (b) The derivation of quantities from the left to forms that we can put in the matrix, i.e. *f*_*ij*_(*x*_*i*_).

### 3.2 Nonlinearities and learning

The plan formulation we described above inherits a number of limitations from the standard input-output model; the first one we will build upon is model linearity. The default model linearity is tremendously problematic—for example there is the implicit assumption which is that labour needs will scale linearly with production demands. To address these issues, a generalisation of the input-output model [30, 37] looks as in Eq 3:

$$\begin{array}{c} (I-F(x))x=d \end{array}$$ (3)

This is profoundly liberating as a proposition, as we can stack production units and have different IO-coeffs values as production scales. We can also extract from individual citizens how important hitting certain targets in their profile is. Solving for *x* now becomes a bit harder, as *F*(*x*) could potentially be any function, but in our case, we constrain it to a specific matrix. Remember that individual columns in the IO matrix represent how much it takes to produce a single unit of output—it makes sense to define the matrix as in Eq 4

$$F\left(x\right)=[\begin{array}{cccc} f_{00}\left(x_{0}\right) & f_{01}\left(x_{0}\right) & \cdots & f_{0n}\left(x_{0}\right)\\ f_{10}\left(x_{1}\right) & f_{11}\left(x_{1}\right) & \cdots & f_{1n}\left(x_{1}\right)\\ \vdots & \vdots & \ddots & \vdots \\ f_{n0}\left(x_{n}\right) & f_{n1}\left(x_{n}\right) & \cdots & f_{nn}\left(x_{n}\right) \end{array}]$$ (4)

Constraining our function to this form has one important benefit; we can ask production units directly how many other goods they need in order to produce certain output units, and data scientists in these facilities can use any machine learning method to “fit” a curve and provide back a function.

When it comes to the actual solution, one can attempt to use the gradient directly. The mean squared error *MSE*((*I* − *F*(*x*))*x*, *d*) has a gradient that is ∇*MSE*((*I* − *F*(*x*))*x*, *d*) = 1/*n*((*I* − *F*(*x*))*x* − *d*)(*I* − *F*(*x*) − *F*′(*x*)*x*), which means that we can solve using any non-linear least squares algorithm—or in fact any other non-linear optimisation algorithm. Another method (that comes from [30]) is to go through the power series expansion ${\left(I-A\right)}^{-1}=\sum_{i=0}^{\infty }A^{i}=I+A+A^{2}+\ldots$. We can then define *x*_(*i*+1)_ = *F*(*x*_(*i*)_)*x*_(*i*)_ + *d*, *x*_(0)_ = *d*—a recursive form of calculating *x*. This is what we are going to use in this paper, as it is based purely on linear solvers, and will find the global maximum as long as convexity is maintained. We could also attempt an end-to-end neural network solution (it is very easy to envision), but there are no (clear) advantages, unless a need arises to model exceptionally complex IO-coeffs while optimising production at the same time, something we are not doing in this paper.

### 3.3 Time and the transition function

When it comes to producing goods and services, a model without a time element is severely limited; real production and consumption obviously have a time dimension. In the case of production, this is expressed in various forms like gestation times, production times, business inventories and depletion of resources. Multiple input-output models that include a time element have been developed [38–40]—for an overview, see [41]. An example of such a model, from [38] is $x\left(t\right)=\sum_{0}^{n}[A_{t+s}\left(-s\right)x\left(t+s\right)]+\sum_{0}^{n}\{B_{t+s+1}\left(-s\right)[x(t+s)+x(t+s+1)]\}+z\left(t\right)$, with *A* matrices representing circulating capital, all *B* matrices representing fixed capital, *z*(*t*) is the demand at each point in time, while −*s* is the ticks before the time *t*. The problem with these models is they were (for the most part) not designed with planning (in the AI sense) in mind. What we need to introduce (as discussed before) is a transition function *T*(*s*′|*s*, *a*) and a notion of state *s*. This can really be anything that makes sense based on the individual components of what we have, but to simplify things we can define state as an inventory indicating how much we hold of everything we have so far, including any unwanted side effects (i.e. externalities) our methods are generating. The transition function now operates on that inventory/externalities vector, by adding things, removing things, showing when something is ready for consumption, and how much needs to be taken to gestation periods.

### 3.4 Plan egalitarianism and externalities

The goal of the plan is to deliver a set of products and services (termed goods in our setup) in real life, so the real rewards can only be measured when the plan has been executed. During the planning phase, however, we should have a reasonable indication of what is the level of rewards we have achieved. Let $\hat{d_{i}}\left(a_{ij}=0\right)$ be the demand for a final good for a certain profile set to zero, with *i* coming from final goods *C*, while *j* coming from profile consumption *P*. When we removed a good from a profile, we generate a surplus. That surplus, divided by how much that profile was expected to get, we define as the egalitarianism of the plan. More formally, in Eq 5 we define egalitarianism ${EG}_{p}$ as

$$\begin{array}{c} {EG}_{p}^{t}=\underset{i\in C,j\in P}{\text{min}}\{(\hat{d_{i}}\left(a_{i,j}=0\right))/(a_{ij}d_{j})\} \end{array}$$ (5)

Every profile created puts certain requirements on the economy in terms of unwanted side effects, commonly referred to as externalities (e.g. carbon from milk and meat production). We model externalities at each point in time as *ρ*(*e*(*x*_*t*_)*x*_*t*_), with the total externalities for a plan being *E*_*p*_—the sum of all externalities in time as in Eq 6, and *ρ* being a function that weights the importance of each externality for each good:

$$\begin{array}{c} E_{p}^{t}=\sum_{0}^{t}\rho (e\left(x_{t}\right)x_{t}) \end{array}$$ (6)

The difference between the way we measure the unwanted side-effects we get versus the goals we achieve is by design. In terms of production goals, a plan is as good as its worst performance. In terms of damage, we are measuring the cumulative effect. A combination of externalities is what underlies the reward function.

### 3.5 Plan execution

Given that we do not have access to the real transition function (akin to training for a robot in an largely imperfect simulation), we suffer from two problems; first, that our plans are as limited in their ability to use future states as the imagination of the model creators. We will try to achieve certain goals every day for a year by following a set of actions that correspond to increasing production, without reference to future states—this is known as open loop planning—and is basically a vector *x* per day. The fact that we re-plan on a daily basis means that we execute the plan in a closed loop setting—so overall we do *open loop planning, closed loop execution* [42, 43]. This is highly reminiscent of methods like Monte Carlo Tree Search [44] that have shown tremendous success in games. The second problem is that the artificial conditions we optimise on might not correspond to reality. Again, this is a common problem in robotics and it is currently attacked by assuming fictional model hyperparameters, as to make the model robust [45].

## 4 Data collection

The real world execution of the plan entails two steps: (a) The planner provides information to the production units on their daily targets and requests information on the previous day history, including IO-coeffs in functional form and externalities. (b) The planner requests information on previous days demand and future demand from each individual (or discovers it).

### 4.1 Production units

Each production unit would have to effectively fill the columns of Matrix *F* by providing the function *f*_*ij*_(*x*_*i*_)*x*_*i*_, This can be achieved trivially by some form of active learning (i.e. asking managers: “how much milk do you need to make one pound of cheese? How about two pounds? How about three?”) and interpolating accordingly. Alternatively, one can seed a classic ML model using past production data and combine it with active learning in any gaps. Now, converting these values into *f*_*ij*_(*x*_*i*_) simply required dividing over the number of actual products *x*_*i*_ for all possible values of *x*_*i*_. We expect production units to innovate constantly, achieving lower externalities and better IO-coeffs, in a very organic process that amounts to optimisation coming from every part of the system.

#### 4.1.1 Citizens

We have defined various profiles, but where do those profiles come from? This is essential—these profiles are our reward function. Learning a reward function from consumption targets can be done by using any form of inverse reinforcement learning/preference learning on existing buying habits, direct questions and/or voting all in accordance with productive capacities. This should allow for effectively the discovery of basic needs on a fundamental level and the provision of relevant goods. From the outset, different profiles aim at addressing the problem of *Variety* [46] directly, i.e. we need to be able to act upon as many world states are possible. Individual profiles for every person would put tremendous strain on the planning mechanism and make the whole system very brittle, as any errors in production will result in a series of complaints. Instead, the focus should be on goods that allow for a high degree of customisation. For example, pre-packaged foods are a really bad production option, as they allow for very little tinkering. Allowing for very high degree of customisation and personalisation (i.g. a combination of (generative?) recipes plus food) should help make production both more robust and interesting. New types of computing devices, whose aim is to help so as to have the goods delivered be used in the most efficient and creative fashion possible, will also prove pivotal.

### 4.2 Interactions with the market

Since the plan’s aim is to complement, rather than abolish, the market, it is worth discussing what areas of production the plan will not shape. Goods in scarcity or products whose only value is their scarcity cannot be delivered through the plan; the subjectivity of the reward function would make it exceptionally hard to calculate individual preferences (and hence profiles), and would also open up the possibility of abuses, requiring constant vigilance to stop the creation of black markets. Goods in scarcity also open questions of multi-objective optimisation [47]—that will mostly lead to a wealth of equally non-satisfactory solutions. Any invention that helps the plan should be readily adapted. New products and services could also come from market forces. This would require the market to turn into activities that look more like prospecting—anything that a plan cannot cover should generate profit. The most important point, however, when it comes to market, is not to allow it to use the plan as a way of undercutting wages; once the plan is introduced, it should be followed by a policy of *increasing minimum wages and decreasing working hours, in accordance with productivity gains* in order to start removing human labour from the market and reaping benefits from further automation. For example, shoe production is still a very manual process, and high wages in the sector should come from automation. This is extremely important, as current trends point to “reverse centaurs” when it comes to automation [48]. Given that it is easier to manage humans using algorithms (e.g. organise taxi drivers) vs performing the actual task (e.g. autonomous cars), there is a certain market tendency to extract profits from over-exploitation. Instead of machines aiding humans (i.e. what is called a “centaur” in chess), we run the risk of economies of very low wages where humans help machines (i.e. more akin to a very boring and intensive production line, hence “reverse centaur’)’.

## 5 Simulations

**Fig 2:**
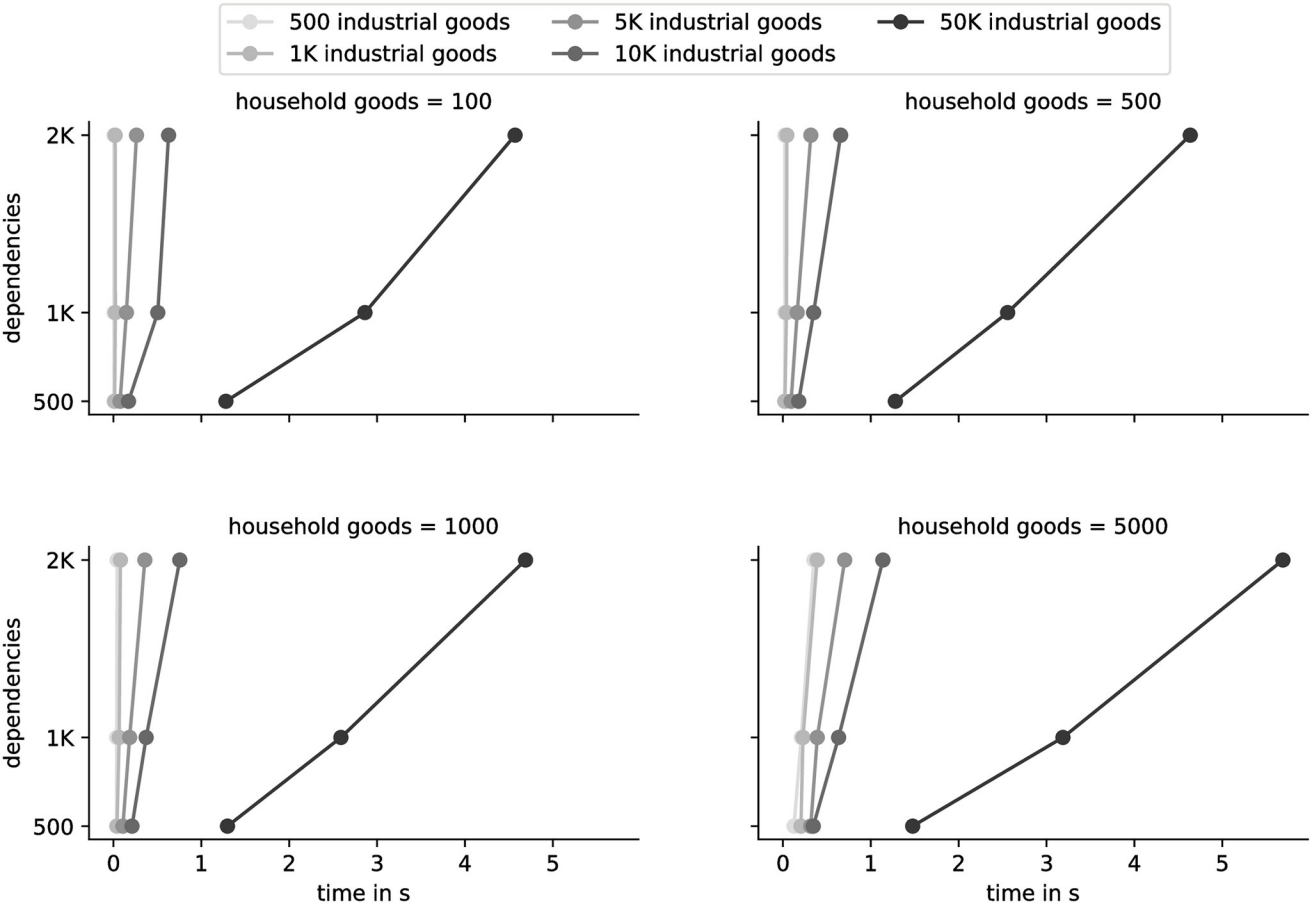
An example of performance scaling in solving the basic equation of our model (*I* − *A*)*x* = *d*. Note that though *A* is sparse, this does not follow that *x* would be.

We performed a number of simulations on imaginary data. The first set of simulations resolves around solving (*I* − *A*)*x* = *d* repeatedly for matrices of different size. Solving this set of linear Equations fast is fundamental as both our time element and the non-linearity solution depend it. We have run all possible combinations of industrial goods (i.e. goods not needed by the profiles, [500, 1000, 5000, 10000, 50000], final goods of [50, 100, 500, 1000, 5000], a profile of size 200 (i.e. 200 different combinations of final goods), with each good needing [500, 1000, 2000] other goods in order to be made. The results can be seen in Fig 2—all results were collected on a CPU: Intel i7-8700K @ 4.800GHz / 64GB RAM, using scipy [49]. Alternative solutions that include gradient estimations might be faster, but this will probably depend on the problem. As it stands, the deciding speed factor is the number of dependencies, but everything is solved in well below 20 seconds. Overall, it is trivial to attack the problem.

**Fig 3:**
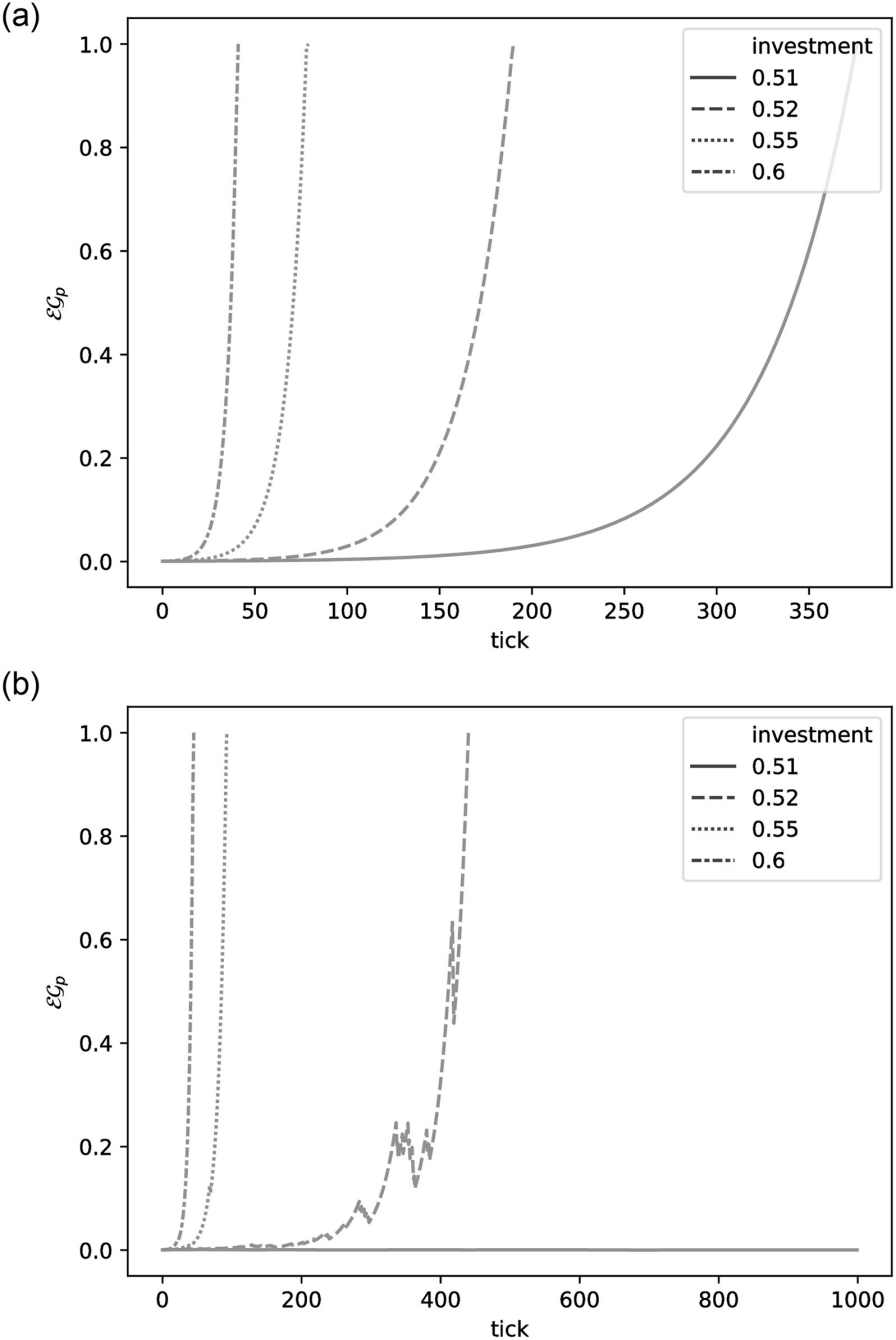
Egalitarianism of the plan vs investment profiles. Note the exponential curves. The x-axis represents “time” ticks, while the y-axis represents the egalitarianism of the plan. (a) A simulation without noise. (b) A simulation with noise—certain investment profiles fail to achieve self-sustainability.

We also simulate a sample, tiny village economy in Table 1. Note that the table is not (exactly) in the form described by Leontief. There is no demand (effectively, it is set to 0) and all consumption is meant to come through the profiles. The economy is made up from two final goods (Milk,Butter) and one industrial good (Butter churn). Butter churn requires a variable amount of Milk to get created (for example, as extra calories for the workers that take part in the process). The initial quantities of each item in inventory are restricted. The results of the simulation can be seen in Fig 3. The village plans to provide the final goods in two profiles. The village starts without being able to fulfil the goals of each profile, hence they are forced to produce a limited amount of goods at each daily tick and invest the rest. What this means in practical terms is that units of Milk we create get “consumed”, while units of Butter churn just get added on. Notice the exponential rise in egalitarianism of the plan. We perform a second experiment, where with a certain probability a portion of the inventory would just vanish. Here (see Fig 3(b)) lower investment leads to collapse, with the egalitarianism of the plan never recovering. This effect would not be visible without including some noise to the model. Finally, also note that the only real difference between a simulation and a plan comes from the fact that we think that the simulation is closer to reality—there is no way to execute it in real life.

## 6 Limitations

We have introduced a planning method based on a non-linear version of input-output tables. Our model is a first attempt to revitalise in-natura economic planning. We identify five major limitations below, alongside potential speculative solutions.

1. Function approximation: all input-output methods treat each individual good and service as unique. This would create an explosion of goods one would need to plan for; for example, every shoe size would be a different type of good, every slightly different health plan a different service and so forth. A common way to address this is through function approximation. All goods of a certain type would be parameterised using a specific feature set, and the problem attacked using algorithms similar to expert iteration [50] or any other modern RL method.
2. Introducing partial observably and stochasticity: the knowledge assumed by the plan so far is far more absolute than one should expect. It would be prudent to assume latent variables in the production/consumption process and general randomness, to account for things that we cannot directly measure. As discussed, this would move the modelling closer POMDP-inspired methods.
3. Lack of realistic simulations: the simulations used in this paper are toy versions compared to what a real world process would look like. As such, they do not allow for a qualitative assessment of the algorithms proposed. In contrast, fields like games have elaborate simulations that allow for exact results and AI has a long tradition of measuring progress this way—see for example [51]. We do not have any such framework here, which would make the measurement of quality of subsequent planning algorithms impossible.
4. Adversarial elements: the current setup implicitly assumes that nobody has an incentive in breaking the plan. There are good reasons to believe that this might not be the case. What is the optimal way of in-natura planning within a (partially) adversarial domain?
5. Sector heuristics: general methods, such as the ones presented here, can greatly improved with the addition of heuristics tailored to the domain under question. Without further field research, such heuristics cannot be uncovered. Thus, a practical application of the methods proposed would require serious research on both household needs and industry production methods.

The above, if seen optimistically, set out a research programme. The combination of function approximation, non-linearity, monte-carlo methods and partial observability would help revolutionise the way we think about our economic future.

## 7 Conclusion

We have presented an in-natura economic planning system, roughly inspired by modern AI developments. It allows for the creation of various consumption profiles and the modelling of arbitrary production capacity of individual production units. As per Section 6, the repeated open-loop planning element mitigates some of the worst problems of missing local information, but the modelling we have done is really basic. We do not capture the sensory issues that would result from the point of view of a decentralised planner adequately (in the form of a belief function) and we have not included any game-theoretic effects in our modelling (e.g. what if a production unit goes rogue and decides to persistently mismanage or misreport its productive capacity?). Overall, we think its time to re-start a research programme on economic planning. We hope that this paper re-starts the discussion on a technical level, with ever increasing planning methods and simulations coming to light. There is no reason for the plan to be as simple as the one discussed here—in fact Facebook is currently performing large scale simulations [52] that include “fake users” and certain corporations use extremely sophisticated systems to manage distribution and production [53]—there is no reason simliar ideas cannot be adopted for a more fair system.
